# UNDERSTANDING THE DIMENSIONS OF SOCIAL RESOURCES AMONG SEXUAL AND GENDER MINORITY OLDER ADULTS

**DOI:** 10.1093/geroni/igac059.1420

**Published:** 2022-12-20

**Authors:** Hyun-Jun Kim, Karen Fredriksen Goldsen, Meghan Romanelli

**Affiliations:** University of Washington, Seattle, Washington, United States; University of Washington, Seattle, Washington, United States; University of Washington, Seattle, Washington, United States

## Abstract

Despite the growing number of recent studies on the positive role of social resources on health and well-being among SGM older adults, the multi-dimensional construct of social resources has not been examined due to a lack of adequate measures and data. This study describes the rationale behind the measurements of social resources used in the National Health, Aging, and Sexuality/Gender Study (NHAS). We analyze NHAS data collected in 2014 (N = 2,450) to assess the reliability of these social resource measurements. Out of 28 indicators of social resources (α = .82), three distinct factor structures emerged: relational social connectedness (α = .71), collective social connectedness (α = .82), and perceived social connectedness (α = .79). All item-rest correlations for each scale were moderate to strong. The scales could be used to understand various aspects of social resources and its role in enhancing health and well-being in the historically marginalized populations.

